# Heteroresistance to clarithromycin and metronidazole in patients with a *Helicobacter pylori* infection: a systematic review and meta-analysis

**DOI:** 10.1186/s12941-022-00509-3

**Published:** 2022-05-20

**Authors:** Ebrahim Kouhsari, Nourkhoda Sadeghifard, Arezoo Khadiv, Hojjat Sayadi, Taghi Amiriani, Sobhan Ghafourian, Hassan Valadbeigi, Marcela Krutova

**Affiliations:** 1 Laboratory Sciences Research Centre, Golestan University of Medical Sciences, Gorgan, Iran; 2 Clinical Microbiology Research Centre, Ilam University of Medical Sciences, Ilam, Iran; 3 Department of Microbiology, Faculty of Medicine, Ilam University of Medical Sciences, Ilam, Iran; 4 Non-Communicable Diseases Research Centre, Ilam University of Medical Sciences, Ilam, Iran; 5 Golestan Research Center of Gastroenterology and Hepatology, Golestan University of Medical Sciences, Gorgan, Iran; 6 Department of Medical Microbiology, Charles University, 2nd Faculty of Medicine and Motol University Hospital, Prague, Czech Republic

## Abstract

**Background:**

Antimicrobial resistance of *H. pylori* can lead to treatment failure. Importantly, several studies have reported on heteroresistance, i.e. the presence of resistant and susceptible *H. pylori* populations in the same sample and/or a difference in the susceptibility patterns between biopsy samples. This meta-analysis aims to provide comprehensive data on the prevalence of metronidazole and clarithromycin heteroresistance and the approaches to their detection.

**Material and methods:**

A systematic review was performed after the search of MEDLINE, Scopus and Web of Science. The study outcomes were the weighted pooled prevalence of heteroresistance to clarithromycin and metronidazole in *H. pylori* positive samples and/or isolates with a subanalysis by continent.

**Results:**

A total of 22 studies that had investigated 3852 *H. pylori* positive patients were included in the meta-analysis. Heteroresistance to clarithromycin was reported in 20 studies, with a weighted pooled prevalence of 6.8% (95% CI 5.1–8.6; 3654 *H. pylori* positive patients; the substantial heterogeneity I^2^ = 55.6%). Heteroresistance to metronidazole was reported in 12 studies, with a weighted pooled prevalence of 13.8% (95% CI 8.9–18.6; 1670 *H. pylori* positive patients; the substantial heterogeneity I^2^ = 60.9%). The weighted pooled prevalence of clarithromycin heteroresistance was similar in Asia and Europe (p = 0.174584), however, metronidazole heteroresistance was detected more often in Europe (p < 0.00001). Clarithromycin heteroresistance was detected more often by phenotype rather than by using genotyping methods (12 vs 8 studies), whereas heteroresistance to metronidazole was detected only by phenotype.

**Conclusion:**

The prevalence of heteroresistance to clarithromycin and/or metronidazole is not negligible and can be detected in approximately 7 and 14% of *H. pylori* positive samples, respectively. These findings highlight the need to raise the awareness of gastroenterologists and microbiologists to the heteroresistance to clarithromycin and metronidazole in patients with a *H. pylori* infection.

**Supplementary Information:**

The online version contains supplementary material available at 10.1186/s12941-022-00509-3.

## Introduction

*Helicobacter pylori,* a gram-negative spiral-shaped bacterium, is one of the most prevalent pathogens worldwide [1]. Peptic ulcer disease, or non-ulcer dyspepsia, are the most common clinical conditions of *H. pylori* infection [2]. *H. pylori* is classified as a group 1 carcinogen that causes gastric adenocarcinoma [3].

*Helicobacter pylori* eradication treatment decreases the incidence of duodenal or gastric ulceration and gastric cancer [2]. A combination of proton pump inhibitors, different antimicrobials and/or bismuth are used for *H. pylori* eradication, however, the increasing antimicrobial resistance can lead to treatment failure [4].

Antimicrobial susceptibility testing for *H. pylori* should be performed using the minimal inhibitory concentration method as recommended by the European Committee on Antimicrobial Susceptibility Testing (EUCAST). However, *H. pylori* is a fastidious organism that requires specific culture conditions [5]. Moreover, a delay in the transport of biopsy samples to a laboratory, or the use of proton pump inhibitors before biopsy, can result in a failure to culture *H. pylori* [6]. To overcome difficulties with *H. pylori* cultures, molecular assays for the detection of *H. pylori* have been developed. In addition to pathogen detection, several assays were designed for the identification of mutations associated with clarithromycin and/or levofloxacin [7].

Metronidazole and clarithromycin are included in non-bismuth quadruple *H. pylori* eradication regimens (concomitant, sequential and hybrid) and in triple therapy when metronidazole can be replaced by amoxicillin. In addition, metronidazole is a part of the bismuth quadruple *H. pylori* eradication regimen; [4] resistance to any of these drugs can lead to treatment failure. The wide spectrum of mechanisms of metronidazole resistance in *H. pylori* have been described, e.g. genetic rearrangements in the *rdxA* gene (insertions and deletions of transposons, and missense and frameshift mutations) and point mutations in the *frxA* and *frxB* genes that can further increase the level of metronidazole resistance in the presence of mutations in the *rdxA* gene [8, 9]. The resistance to clarithromycin is caused by point mutations in the 23S ribosomal subunit (23S rRNA). Four conserved efflux systems families have been also described in *H. pylori* strains [10].

In addition to resistance, the occurrence of heteroresistance in *H. pylori* isolates or samples has been reported [11]. Heteroresistance, a mixture of susceptible and resistant patterns, was found in *H. pylori* isolates and/or samples from the same site of biopsy (intraniche) or from *H. pylori* isolates and/or samples from different biopsy sites (interniche) [12]. Interestingly, heteroresistant *H. pylori* causative strains demonstrate similar fingerprinting patterns [13–17] suggesting the presence of the same strain with and without resistance mechanisms (monoclonal heteroresistance) rather than a co-infection with different strains (polyclonal heteroresistance) [11].

This study aims to summarize data on the prevalence of metronidazole and clarithromycin heteroresistance and the approaches to their detection.

## Methods

### Search strategy and study selection

Three databases including MEDLINE [PubMed], Scopus, and Web of Science were searched for relevant articles (Up to February 3rd, 2020) by using the following keywords: “*Helicobacter pylori”* OR *“H. pylori”* AND “heterogeneous resistance” OR “resistance heterogeneity” OR “heteroresistance” OR “antimicrobial heteroresistance” OR “metronidazole heteroresistance” OR “clarithromycin heteroresistance” in the Title/Abstract/Keywords fields. Only studies written in English were included. Reference lists of all related studies were also reviewed for any other related publications. The records found by searching the database were merged and the duplicates were removed using EndNote X7 (Thomson Reuters, New York, NY, USA).

### Selection criteria and data extraction

The information extracted from each study included: (1) first author; (2) publication year; (3), patient gender and age (mean, range, paediatrics vs. adults); (4) biopsy site; (5) number of samples*;* (6) the method of heteroresistance detection; (7) heteroresistance rates; and (8) a definition of heteroresistance. A summary of the extracted data is shown in the Additional file 1: Table S1.

Exclusion criteria were: (1) heteroresistance was not detected; (2) the results of heteroresistance were not clearly reported; and (3) data on heteroresistance were from a meta-analysis and/or systematic review, non-original research or conference abstract.

### Statistical analysis

Studies presenting data on metronidazole and/or clarithromycin heteroresistance were included in the meta-analysis which was performed by computing the pooled prevalence of heteroresistance for each antimicrobial agent using a random-effects model [18]. Inconsistencies across studies were examined by the forest plot as well as the I^2^ statistic. Values of I^2^ (25%, 50% and 75%) were interpreted as the presence of low, medium, or high heterogeneity, respectively.

### Study outcomes

The main outcome of the study was the weighted pooled prevalence of heteroresistance to clarithromycin and metronidazole with subgroup analysis for the continent (Asia and Europe).

## Results

**Fig. 1:**
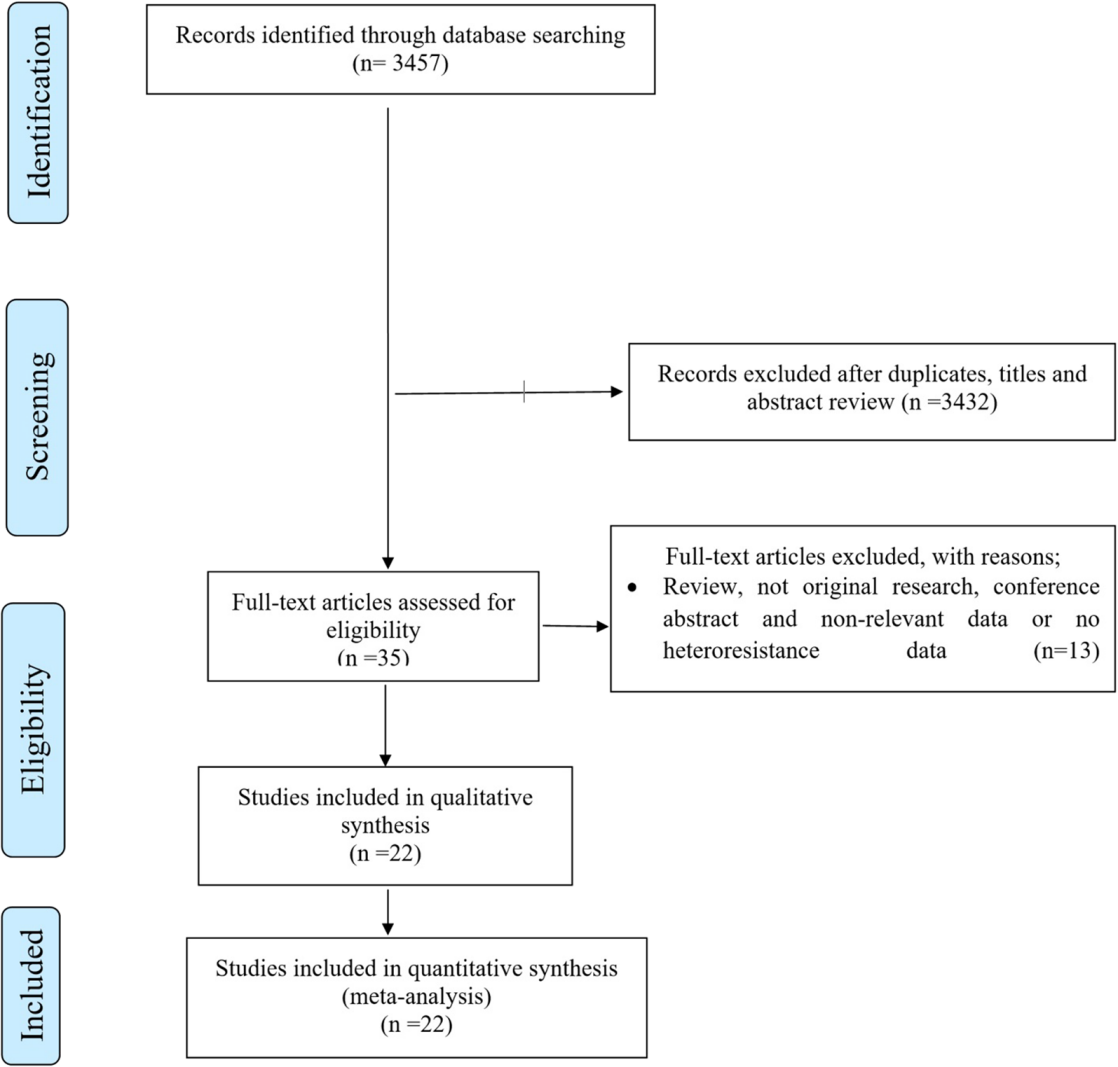
Flow chart of study selection

A total of 3457 records were identified in the initial search. From these, 3432 articles were excluded after an initial screening of the title and abstract due to their irrelevance or duplication. The full texts of the remaining 35 articles were reviewed. From the 35 articles, 13 were excluded for the following reasons: reviews; non-original researches; conference abstract; and non-relevant data or that no heteroresistance data were reported. Finally, 22 studies were included in this systematic review and meta-analysis (Fig. 1, Additional file 1) [12–17, 19–34].

In 22 studies, 3852 *H. pylori* positive patients were investigated. According to the continent, the majority of studies were from Europe (n = 10, 2172 patients), followed by Asia (n = 7, 1331 patients), America (Argentina, Mexico and Colombia 195 patients, Africa (Tunisia, 21 patients) and the Middle East (Turkey and Iran, 133 patients), Table 1, Aditional File 1.

**Table 1:** Studies detecting heteroresistance to metronidazole and/or clarithromycin in *Helicobacter pylori* isolates/positive samples

Study	Country	*H. pylori*-positive patients	Testing method	MTZ_R	MTZ_HR	CLR_R	CLR_HR
Kocsmár et al., 2020 [12]	Hungary	305	rRNA‐targeted FISH	NA	NA	35	38
Güven et al., 2019 [19]	Turkey	93	HelicoDR test	NA	NA	22	12
Farzi et al., 2019 [16]	Iran	40	Sequencing of 23S rDNA	NA	NA	7	7
Arévalo-Jaimes et al., 2019 [17]	Colombia	63	Sequencing 23S rDNA	NA	NA	19	5
Sun et al., 2018 [20]	China	49	Droplet digital PCR	NA	NA	11	13
Mascellino et al., 2018 [21]	Italy	40 (30)	E-test, Real-Time PCR	15	1	15	4
Aguilera-Correa et al., 2017 [22]	Spain	111	HelicoDR test	NA	NA	53	11
Mansour et al., 2016 [23]	Tunisia, France	42	E-test	21	7	12	6
Navarro-Jarabo et al., 2015 [24]	Spain	401	HelicoDR test	NA	NA	35	37
Kao et al., 2014 [14]	Taiwan	412	E-test	NA	16	NA	1
Selgrad et al., 2014 [25]	Germany	66	E-test	30	4	27	2
Ayala et al., 2011 [26]	Mexico	90	E-test	47	17	4	5
Marzio et al., 2011 [27]	Italy	68	Agar dilution	NA	NA	12	15
Norazah et al., 2009 [28]	Malaysia	22	E-test	6	6	0	1
Matteo et al., 2006 [15]	Argentina	42	Agar dilution	6	8	NA	NA
Raymond et al., 2005 [29]	France	28	E-test	22	14	13	3
Lee et al., 2005 [30]	South Korea	21	Agar dilution	4	12	2	5
Rimbara et al., 2005 [31]	Japan	542 (541)	Agar dilution	4	4	39	25
Kim et al., 2003 [13]	South Korea	220	Agar dilution	72	28	7	6
Masuda et al., 2003 [32]	Japan	65	PCR-SSCP, sequencing	NA	NA	5	11
van der Ende et al., 2001 [33]	Netherlands	976	E-test	NA	NA	45	6
Weel et al., 1996 [34]	Netherlands	156	Disk diffusion test, E-test	37	52	NA	NA

The number in brackets indicates the number of samples investigated for metronidazole resistance when it differed from the number of samples investigated for clarithromycin resistance

*R* resistance (samples/isolates with heteroresistance are not included), *HR* heteroresistance

**Fig. 2:**
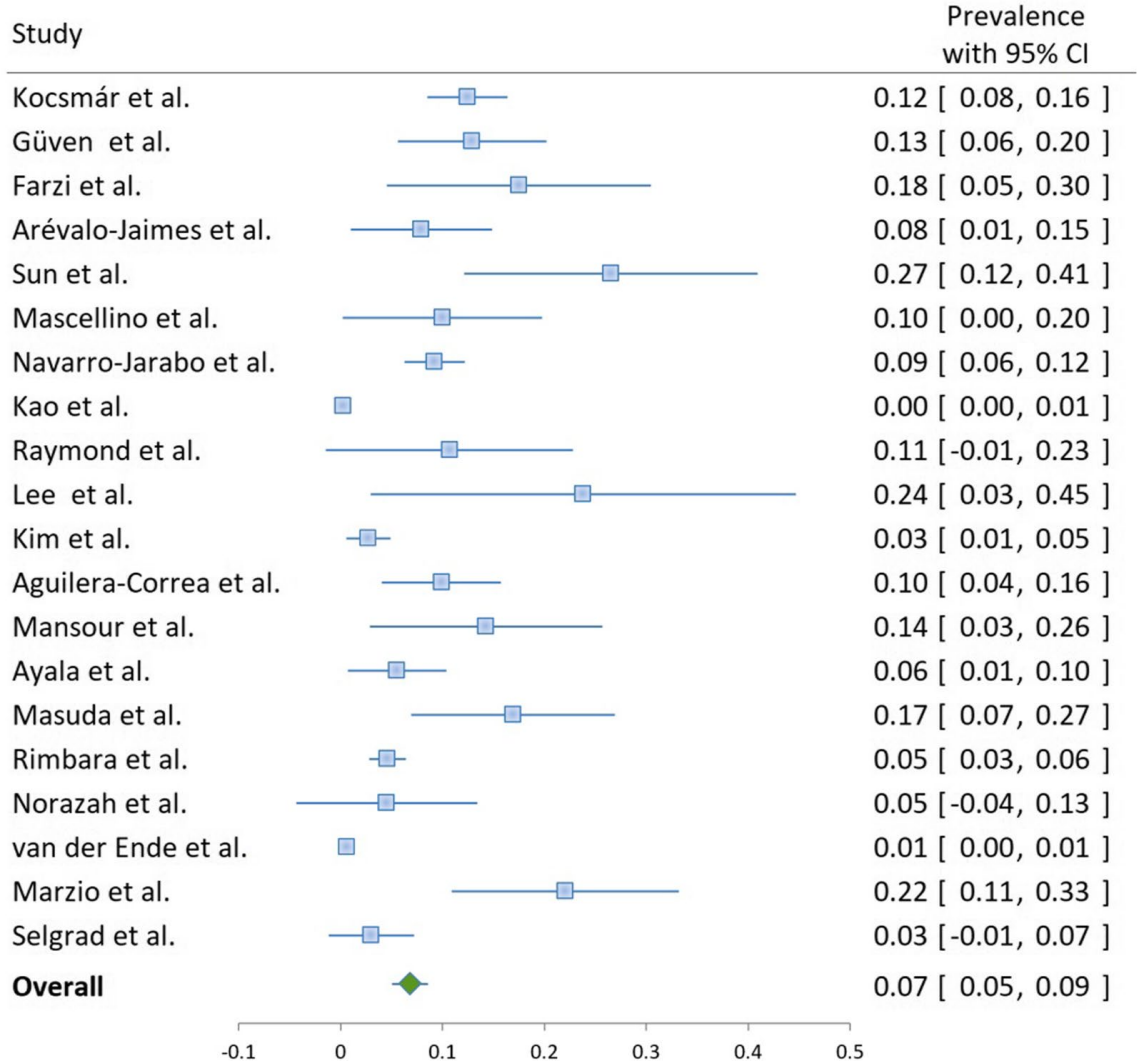
Clarithromycin resistance in *Helicobacter pylori*-positive samples or isolates

**Fig. 3:**
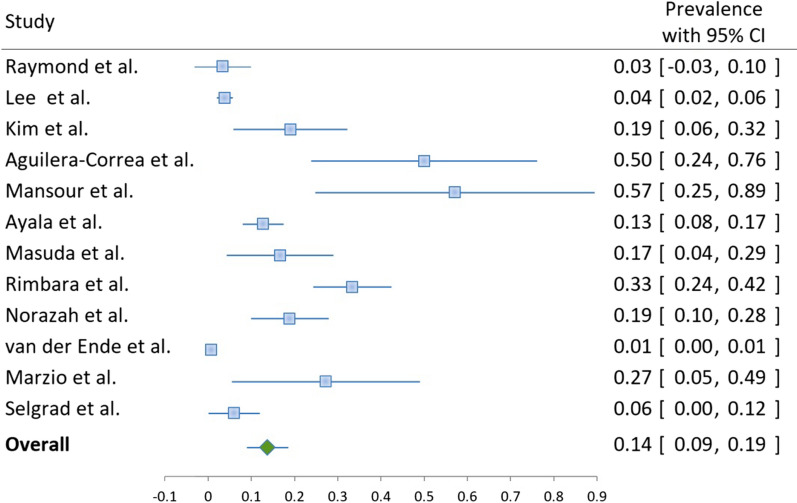
Metronidazole resistance in *Helicobacter pylori*-positive samples or isolates

Heteroresistance to clarithromycin was reported in 20 studies, with a weighted pooled prevalence of 6.8% (95% CI 5.1–8.6) among 3654 *H. pylori* positive patients; the substantial heterogeneity was I^2^ = 55.6%. (Table 1, Fig. 2). Heteroresistance to metronidazole was reported in 12 studies, with a weighted pooled prevalence of 13.8% (95% CI 8.9–18.6) among 1670 *H. pylori* positive patients and substantial heterogeneity (I^2^ = 60.9%) (Table 1, Fig. 3).

The weighted pooled prevalence of clarithromycin heteroresistance was similar in Europe (8.4%; 95% CI 3.8–12.9%; I^2^ = 0), and Asia (5.6%; 2.1–9.1%; I^2^ = 61.8%, p-value 0.174584); however, when compared to Asia, the metronidazole heteroresistance was detected more often in European *H. pylori* positive patients (19.6%; 95% CI 5.6–33.6%; I^2^ = 24.7% vs. (7.6%; 95% CI 2.4–12.8%; I^2^ = 73.3%), Additional files 2, 3, 4, 5: Fig. S1-S4. Data for the Middle East, Africa and America were not calculated due to the small number of studies.

Clarithromycin heteroresistance was detected by phenotype in 12 studies (agar dilution n = 5, E-test n = 7) and by genotype in eight studies (Table 1). Three studies used the same commercial molecular assay HelicoDR test (Hain Lifescience, Germany). In the study of Navarro-Jarabo et al., this assay was applied to *H. pylori* isolates and in the studies of Aguillera-Correa et al. and Güven et al., DNA extracted from biopsy samples was tested [19, 22, 24]. Another commercial assay, BACTfish *H. pylori* Combi kit (Izinta Kft., Hungary) was used to analyse the biopsy specimens [12]. Two other studies designed their molecular assay: one was based on Real-Time PCR followed by a melting curve analysis using fluorochrome-labelled probes in DNA from *H. pylori* isolates; the second analysed DNA from gastric brushes samples using droplet digital PCR [20, 21]. In contrast to the heteroresistance to clarithromycin, the heteroresistance to metronidazole was detected only by phenotype (agar dilution n = 4, E-test n = 7, disk diffusion followed by E-test n = 1), (Table 1).

## Discussion

Antimicrobial susceptibility testing is essential for the administration of effective antibiotic treatment and the control of antimicrobial resistance, however, antimicrobial susceptibility testing in causative *H. pylori* strains is recommended after second-line treatment failure. Given that a combination of antimicrobials is used for the treatment of *H. pylori* infections, antimicrobial susceptibility testing should be performed to reduce the burden on the patient and decrease the risk of eradication treatment failure through the administration of ineffective antimicrobial drugs [35].

Global data gathered by the World Health Organization (WHO) on the resistance of antimicrobials used for the eradication of *H. pylori* show an alarming upward trend in all WHO regions [36]. In addition to resistance, the occurrence of heteroresistance in *H. pylori* isolates or samples has been described [11]. The heteroresistance can be detected intraniche by the presence of susceptible and resistant patterns in one strain and/or sample and interniche when differences in susceptibility patterns are observed between strains and/or samples from different biopsy sites [12]. As was shown, the interniche heteroresistance can be undetected in one-fifth of cases when only one antrum biopsy site approach is used [12]. Two biopsy sites, where preferably multiple biopsies are taken, can increase the probability of differences in antimicrobial susceptibility patterns [12].

In our meta-analysis that included 22 studies, a weighted pooled prevalence of heteroresistance to clarithromycin was 6.8% (95% CI 5.1–8.6) and heteroresistance to metronidazole was shown to be greater than two times higher at 13.8% (95% CI 8.9–18.6). These data are consistent with the latest WHO data on resistance of *H. pylori* where resistance to metronidazole was found to occur approximately twice as often as resistance to clarithromycin in all WHO regions, except for the Americas [36].

Interestingly, in several studies, the heteroresistant phenotype was detected rarely in several isolates [25], however, other studies have shown an equal or even higher number of heteroresistant samples compared to resistant phenotype [12, 24, 27].

The subgroups analysis of the methods for heteroresistance showed that a majority of studies detected heteroresistance by phenotype; E-test was the most common. Recently, the E-test performance was compared to agar dilution on 72 clinical *H. pylori* isolates and a high essential agreement (> 90.0%) was found for amoxicillin, erythromycin, tetracycline and levofloxacin, but it was only 84.7% for metronidazole. However, higher detected rates of resistance by the E-test were not statistically significant [37].

Genotyping methods were used for the detection of mutations in the 23S rRNA gene associated with resistance to clarithromycin [9]. In our meta-analysis, four studies used a commercial molecular assay [12, 19, 22, 24]. Two other studies used lab-developed molecular assays [20, 21]. None of the studies used a genotyping method for the detection of heteroresistance to metronidazole probably due to the nature of the molecular mechanisms. The wide spectrum of genetic changes in the *rdxA,* the main mechanisms of resistance to metronidazole, complicates the design of a molecular assay and, for now, the detection of resistance to metronidazole relies on phenotype-based susceptibility testing [9].

## Conclusion

The prevalence of heteroresistance to clarithromycin and/or metronidazole is not negligible and can be detected approximately in 7 and 14% of *H. pylori* positive samples, respectively. These findings highlight the need to raise the awareness of gastroenterologists and microbiologists to the heteroresistance to clarithromycin and metronidazole in patients with a *H. pylori* infection. Therefore, data on heteroresistance should be included in a new guidance document for the diagnosis and treatment of *H. pylori* infections [38]. This meta-analysis can serve as solid evidence for this purpose.

## Supplementary Information

**Additional file 1. **Summary of included studies.

**Additional file 2:** **Figure S1.** Clarithromycin resistance in *Helicobacter pylori*-positive samples/isolates in Europe.

**Additional file 3: Figure S2.** Clarithromycin resistance in *Helicobacter pylori*-positive samples or isolates in Asia.

**Additional file 4: Figure S3.** Metronidazole resistance in *Helicobacter pylori*-positive samples or isolates in Europe.

**Additional file 5: Figure S4**. Metronidazole resistance in *Helicobacter pylori*-positive samples or isolates in Asia.

## Data Availability

Not applicable.

## Abbreviations

EUCAST: European Committee on Antimicrobial Susceptibility Testing
23S rRNA: 23S ribosomal subunit
WHO: World Health Organization
