# Supplementary material for: Heteroresistance to clarithromycin and metronidazole in patients with a Helicobacter pylori infection: a systematic review and meta-analysis
Source: Ann Clin Microbiol Antimicrob. 2022 May 20;21:19. doi: 10.1186/s12941-022-00509-3 (PMC9123761; doi:10.1186/s12941-022-00509-3)
Supplement: Supplementary file 2 — Additional file 2: Figure S1. Clarithromycin resistance in Helicobacter pylori-positive samples/isolates in Europe. [file 12941_2022_509_MOESM2_ESM.docx]

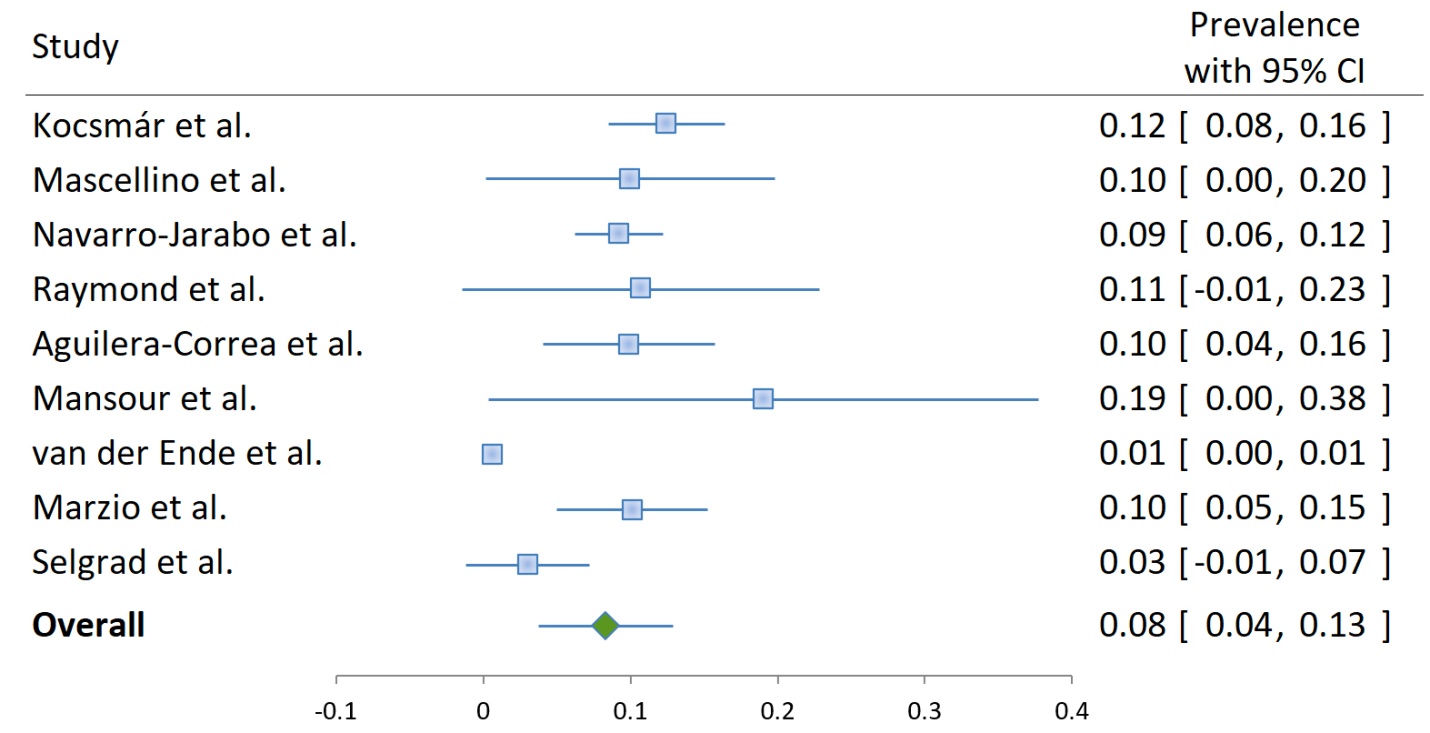


Figure S1: Clarithromycin resistance in *Helicobacter pylori*-positive samples/isolates in Europe.
