# Supplementary material for: Heteroresistance to clarithromycin and metronidazole in patients with a Helicobacter pylori infection: a systematic review and meta-analysis
Source: Ann Clin Microbiol Antimicrob. 2022 May 20;21:19. doi: 10.1186/s12941-022-00509-3 (PMC9123761; doi:10.1186/s12941-022-00509-3)
Supplement: Supplementary file 5 — Additional file 5: Figure S4. Metronidazole resistance in Helicobacter pylori-positive samples or isolates in Asia. [file 12941_2022_509_MOESM5_ESM.docx]

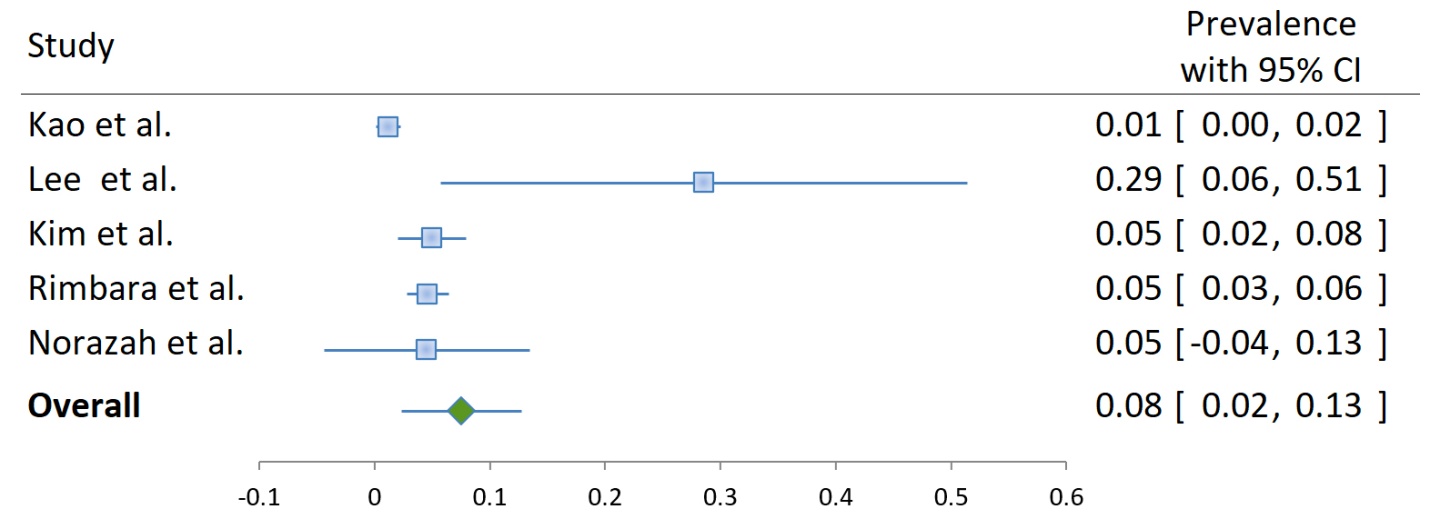


Figure S4: Metronidazole resistance in *Helicobacter pylori*-positive samples or isolates in Asia
